# The Risk Behaviors and Mental Health of Detained Adolescents: A Controlled, Prospective Longitudinal Study

**DOI:** 10.1371/journal.pone.0037199

**Published:** 2012-05-18

**Authors:** Zhenhua Zhou, Hongyan Xiong, Ran Jia, Guoyu Yang, Tianyou Guo, Zhaoyou Meng, Guangyu Huang, Yao Zhang

**Affiliations:** 1 Department of Neurology, Laboratory for Brain and Cognitive Sciences, Southwest Hospital, Third Military Medical University, Chongqing, China; 2 Department of Epidemiology, College of Preventive Medicine, Third Military Medical University, Chongqing, China; 3 Department of Psychological Medicine and Psychiatry, Third Military Medical University, Chongqing, China; 4 Department of Psychology, Shenzhen University, Shenzhen, China; 5 Institute of Infectious Diseases, Southwest Hospital. Third Military Medical University, Chongqing, China; The University of Queensland, Australia

## Abstract

**Background:**

To assess the behavioral risk factors and mental health needs of adolescents in juvenile detention centers (JDC).

**Method:**

A total of 238 boys aged 12–17 years was surveyed who had been admitted to a detention center and compared them with boys from the community (n = 238) matched for sex and age. We assessed behavioral risk factors and mental health problems by using the Youth Risk Behavior Survey questionnaire (YRBS) and the Youth Self-Report questionnaire (YSR).

**Results:**

Young offenders had significantly higher YRBS scores than controls for drug use (odds ratio (OR) 5.16, 95% CI 2.27–7.84), sexual intercourse (OR, 2.51; 95% CI 1.55–2.90), irregular diet (4.78, 2.11–7.51), suicide attempts (1.96, 1.32–5.85), and physical fighting behavior (3.49, 1.60–7.07), but not for tobacco use, alcohol use, and high–risk cycling. Young offenders at the time of admission (6.61, 2.58–15.2), at 6 months (3.12, 1.81–10.1), and at 12 months (5.29, 1.98–13.3) reported statistically higher levels of total mental health problems than adolescents in a community sample.

**Conclusions:**

Young offenders have a high rate of mental and behavioral disorders. In the detention period, aggressive behavior, self–destructive/identity, and externalizing of problems improved while withdrawn, anxious or depressed, and internalizing of problems worsened.

## Introduction

The few young people who commit criminal offenses require some kind of secure detention. In China, the type of secure provision for young offenders is called a juvenile detention center. The detention center is a security facility designed to house juveniles who need to be detained in a restrictive environment. The institutions are run by the prison service, and places for children aged 14–17 years are managed by the local justice bureau. Programs are scheduled for all youth in the detention center and consist of education, regular exercise, recreational activities, and physical labor for public welfare until the youth are released from detention [1], [2].

Because of the risk that young offenders pose to themselves through problematic substance use, risky sexual behavior or suicide, the psychosocial and health problems of these adolescents cause great public concern worldwide [3], [4]. There is now considerable evidence that young offenders are at an increased risk of mental health problems. The findings from previous research span a range of disorders, including anxiety, depression, social phobia, post-traumatic stress disorder and suicidal behavior [5], [6]. In addition to mental health problems, such youngsters also have been shown to be at an increased risk of behavioral factors, such as excessive drinking, smoking, poor learning, premature love and internet addiction [7], [8]. Despite the growing evidence that young offenders are at risk of a range of mental health problems, few studies have made direct comparisons with non-offender samples [9]. Furthermore, most previous research on young offenders has tended to be cross-sectional, and there has been little systematic research on their mental needs using a longitudinal study [10], [11].

In order to assess a broad spectrum of behavioral and psychological problems in adolescents, a self-administered questionnaire was used to obtain information from the adolescents themselves to assess health-risk behaviors and emotional syndromes. Our aim was to measure the difference in health behaviors and mental health status between juvenile delinquents and general adolescents from the community. The study specifically aimed to measure youth mental health needs at 1 week, 6 months, and 12 months after they were admitted to a detention center. From our longitudinal observational data, we assessed how needs changed over time and the interventions that should be used for different detention periods.

## Methods

### Participants

This control study includes 263 juvenile offenders (all boys) who were convicted and serving sentences of at least 12 months under the supervision of the juvenile detention center in Chongqing, China. Of these youth, 11 refused to participate, 8 withdrew from the study, and 6 were excluded because of incomplete answers. We excluded detained youth who were not 14–17 years old, those of a race/ethnicity other than Asian, and youth that were detained longer than 1 week before the first survey (to minimize recall bias); the remaining 238 juvenile offenders were evaluated (response rate 90.5%).

The control group was randomly selected from households registered at their local residents’ committee that were in the same community at the offender sample. These adolescents were sent a letter inviting them to participate in the study, followed by a telephone call a week later. Additionally, a full explanation of the assessment and study procedure was given to adolescents and families in the letter. Participants with missing information for age, gender, or race/ethnicity were excluded. Finally, the community controls (n = 238) were matched for sex (all boys) and age (14–17 years old). None of the adolescents in the control group had been supervised by the JDC.

### Procedures

The survey was conducted by the United Nations Children’s Fund (UNICEF) to provide comprehensive data with regard to health behaviors, attitudes, and mental health status. Approval from the ethics committee at The Third Military Medical University was obtained, and parents (guardians) gave their written informed consent before entering the study. Data collection occurred from May 2006 to September 2009. All of the interviewers attended a special training seminar organized by the first author of the study. Firstly, they knew the objective of this study. Secondly, they grasp the study procedure and their own major work. Thirdly, highlight special concerns during this study were provided to all of the interviewers. The offender samples completed the self-administered questionnaire in a JDC classroom, and the community samples completed the self-administered questionnaire in their community centers by trained research assistants. To preserve confidentiality, the study data were provided in anonymous form. During the initial intake interview, informed consent was obtained from the parents or guardians because all participants were younger than 18 years old. After we received consent, the study was carried out in two parts. First, the teens participated in a 30-minute interview with a trained research assistant. The interview included demographic questions, questions regarding social support, family background, household incomes, and educational levels. Next, all participants completed three self-administered questionnaires, which include questions regarding demographic information, behavioral problems and mental health problems. Mental health problems of the young offenders should be assessed based on long periods of observation. Therefore, the YSR was administered three times. The first assessment took place the first week they were admitted to the detention center. The second assessment was conducted 6 months later, and the third assessment was conducted 12 months later. In most cases, the questionnaires were completed in less than 60 minutes.

### Measures

The questionnaire consisted of three self-administered instruments to complete. The instruments for this study were the demographic questionnaire, YRBS [12], and YSR [13]. The demographic questionnaire collected information regarding sex, age, regional distribution, education, household income, family structure and religious preference. The remaining two instruments were as follows.

All items measuring health–risk behaviors were adapted from the questionnaire of the YRBS developed by the Centers for Disease Control and Prevention (CDC) in the USA, which has been widely used in adolescents to monitor the prevalence of behaviors that most influence health. All the items are scored on a 3-point Likert scale: 0 (*never*), 1 (*sometimes*), and 2 (*often*). These health-risk behaviors include tobacco use (past 30 days), alcohol use (past 30 days), drug use (past 3 months), sexual behaviors (past 3 months), irregular diet (past 3 months), high–risk cycling (past 12 months), suicide attempts (past 12 months), and physical fights (past 12 months). Detailed information about the survey methodology in the YRBS can be found elsewhere [14]. Its reliability and validity has been well demonstrated in the present study (Cronbach’s α range 0.79–0.86).

The youth self–report questionnaire was used to assess the presence of mental health problems among the participants. It has been widely used and has shown high reliability and validity when used as screening tests for mental health problems of teenagers. The instrument comprises 112 items scored on a 3-point Likert scale: 0 (*not true*), 1 (*somewhat or sometimes true*), and 2 (*very true or often true*), according to how well they describe themselves in the past 6 months. By summing the scores on all items, nine syndromes are diagnosed withdrawn, somatic complaints, anxiety/depression, social problems, thought problems, attention problems, delinquent behavior, aggressive behavior and self-destructive/identity problems. Additionally, scores for internalizing problems, externalizing problems and total problems were also calculated [15]. The internal consistency reliability was high for the juvenile offenders group and the control group (Cronbach’s α range 0.76–0.89).

### Data Analysis

We used EpiData 3.1 (The EpiData Association) to enter the data and STATA release 8.0 (Stata Corporation, College Station, TX) for the analysis. Descriptive statistics were calculated including frequencies for categorical data and means ±SD (standard deviation) for continuous data. A student’s t-test and Chi-square statistics were used to compare the demographic characteristics of participants. Fisher’s exact tests were used when the expected counts were less than five. We compared health–risk behaviors and mental health to look for changes between young offenders and the community group using conditional logistic regression models to calculate the odds ratios and a 95% confidence (95% CI) interval, while adjusting for demographic?characteristics that were significantly different (age, education level, and family type). We assessed whether the changes were due to exposure to the incarceration environment and compared changes in YSR scores between groups using logistic regression. All p values are two–sided, and values less than.05 were generally considered statistically significant in the analyses.

## Results

A description of demographic characteristics is presented for two groups in Table 1. A total of 476 boys completed the questionnaire of which 238 were from the detention center and 238 were sex- and age-matched controls from the community. The average age of the young offender sample was 16.6 years (SD = 1.1), and the average age of adolescents in the community was 16.0 years (SD = 0.9). In general, young offenders were more likely to have a low–level of education, to come from single-parent households, and to live separately from their family as compared with community adolescents (all p values<0.05). We also analyzed whether household income, religious belief, and ethnicity differed between the two groups, but none of these characteristics were significant (range of p values: 0.11–0.50).

**Table 1:** Demographic characteristics of participants.

	Young offenderSample (n = 238)	CommunitySample (n = 238)	P
Mean (95% CI) age	16.62(15.18–17.74)	16.03 (15.15–17.33)	0.013
In low–level education (Primary or secondary school)	113(47.5%)	47(19.8%)	<0.0001
In low–income family (<average level)	91(38.2%)	80(33.6%)	0.19
Single–parent family	40(16.8%)	21(8.8%)	0.02
Living with family	205(86.1%)	220(92.4%)	0.04
Buddhist beliefs	44(18.5%)	56(23.5%)	0.21
Other religion	3(1.2%)	7(2.9%)	0.50
Ethnicity (Han people)	224(94.1%)	232(97.5%)	0.11

The proportion of young offenders with behavioral risk factors, as measured by the YRBS, was higher than in community adolescents (Table 2). The logistic regression also showed the same pattern. For all behavioral risk factors, odds ratios of young offenders were greater than 1.0 compared to community adolescents, and all p values were less than 0.05 except for alcohol use (p = 0.220) and high–risk cycling (p = 0.141). The adjusted odds ratios showed that young offenders had significantly higher YRBS scores than controls for drug use (odds ratio 5.16, 95% CI 2.27–7.84), sexual intercourse (2.51, 1.55–2.90), irregular diet (4.78, 2.11–7.51), suicide attempts (1.96,1.32–5.85), and physical fights behaviors (3.49, 1.60–7.07) but not for tobacco use, alcohol use, and high–risk cycling.

**Table 2:** Outcomes for behavioral problems reported on the YRBS.

	Young offender(n = 238)	Control(n = 238)	Odds ratio(95% CI)	Adjusted odds ratio(95%CI)†
Tobacco use	81(34.0%)	57(23.9%)	1.81(1.06–2.72)	1.70(0.92–2.41)
Alcohol use	74(31.1%)	55(23.1%)	1.54(0.90–2.94)	1.28(0.63–2.59)
Drug use	48(20.2%)	11(4.6%)	5.22(2.45–8.02)	5.16(2.27–7.84)
Sexual intercourse	89(37.4%)	52(22.0%)	2.13(1.21–3.53)	2.51(1.55–2.90)
Irregular diet	77(32.4%)	20(8.4%)	5.05(2.59–7.83)	4.78(2.11–7.51)
High–risk cycling	95(39.9%)	73(30.7%)	1.40(0.71–3.26)	1.09(0.43–2.27)
Suicide attempts	36(15.1%)	14(5.9%)	2.57(1.46–6.38)	1.96(1.32–5.85)
Physical fighting behavior	121(50.8%)	52(21.8%)	3.62(1.85–7.32)	3.49(1.60–7.07)

Young offenders at the time of admission (6.61, 2.58–15.2), 6 months (3.12, 1.81–10.1), and 12 months later (5.29, 1.98–13.3) reported more total mental health problems at a statistically significant level than adolescents in the community sample (Table 3). After entering the detention center, there was a great reduction in the overall amount of problems. For example, the percentage of delinquent behavior in the first interview was 38.2%, whereas in the second and the third interviews it was 13.0% and 11.3%, respectively. However, there were some factors where the frequency of the problem was reduced, but the overall level remained high. For instance, the social problems (9.7%) and thought problems (10.5%) were significantly higher than controls in the third interview. The adjusted logistic regression showed that the aggressive behavior, self-destructive/identity problems and externalizing problems were substantially closer to the controls when 6 months after admission (p values 0.082, 0.154 and 0.069, respectively), as did delinquent behavior after 12 months (p value 0.132).

**Table 3:** Outcomes for mental health problems reported on the YSR.

YSR syndrome	Young offender (n = 238)				Control(n = 238)		
	First interview		Second interview		Third interview		
	Prevalence	Odds ratio†(95%CI)	Prevalence	Odds ratio†(95%CI)	Prevalence	Odds ratio†(95%CI)	Prevalence
Withdrawn	41(17.2%)	5.26(2.48–9.67)	39(16.4%)	2.57(1.31–4.54)	59(24.8%)	2.95(1.44–6.79)	13(5.4%)
Somatic complaints	26(10.9%)	2.31(1.11–5.47)	29(12.2%)	2.22(1.08–6.16)	27(11.3%)	1.86(1.15–5.18)	12(5.0%)
Anxious or depressed	43(18.1%)	5.13(1.71–10.8)	35(14.7%)	3.58(1.40–8.62)	56(23.5%)	5.52(2.29–13.5)	13(5.4%)
Social problems	28(11.8%)	3.16(1.48–7.62)	26(10.9%)	2.68(1.28–5.27)	23(9.7%)	1.91(1.06–4.29)	10(4.2%)
Thought problems	36(15.1%)	3.94(2.02–8.17)	32(13.4%)	3.25(1.68–7.13)	25(10.5%)	2.33(1.21–6.05)	9(3.8%)
Attention problems	33(13.9%)	3.22(1.77–11.1)	34(14.3%)	3.14(1.81–9.46)	30(12.6%)	2.80(1.56–9.01)	12(5.0%)
Delinquent behavior	91(38.2%)	8.83(3.50–17.4)	31(13.0%)	1.62(1.03–7.52)	27(11.3%)	1.36(0.62–7.45)*	17(7.1%)
Aggressive behavior	72(30.3%)	6.91(3.63–20.5)	25(10.5%)	2.01(0.89–13.0)*	22(9.2%)	1.73(0.71–14.6)*	13(5.4%)
self destructive or identity problems	29(12.2%)	3.38(1.29–9.76)	17(7.1%)	1.75(0.43–8.84)*	14(5.9%)	1.51(0.36–9.12)*	9(3.8%)
Internalizing problems	101(42.4%)	5.04(2.33–13.9)	95(39.9%)	4.89(2.08–14.2)	126(52.9%)	6.65(2.79–16.6)	32(13.4%)
Externalizing problems	137(57.6%)	8.25(3.28–26.3)	51(21.4%)	2.07(0.96–7.66)*	46(19.3%)	1.61(0.52–8.13)*	31(13.0%)
Total problems	132(55.5%)	6.61(2.58–15.2)	97(40.7%)	3.12(1.81–10.1)	116(48.7%)	5.29(1.98–13.3)	36(15.1%)

**Figure 1:**
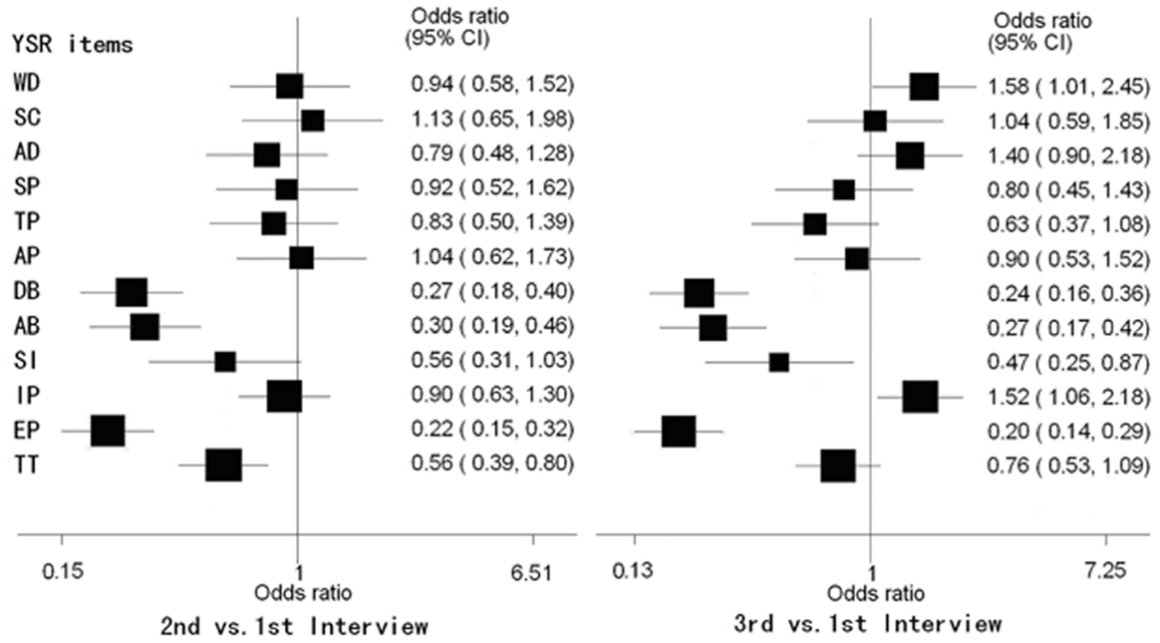
Comparison of YSR scores from the second and third interviews with the first.

The figure compares YSR scores from the second and third interviews with the first, including the odds ratios and 95% confidence intervals (Fig. 1). After admission to JDC, there was a great reduction in several domains. For example, there were large improvements in delinquent behavior, aggressive behavior, externalizing problems, and total problems between the initial interview and the 6-month interview, which were also found in the third interview except for total problems. However, in contrast with the second interview, there was an obvious increase in the frequency of withdrawn (1.67; 95% CI, 1.07 to 2.60; p = 0.031), anxious or depressed behavior (1.77; 95% CI, 1.12 to 2.79; p = 0.020), and internalization of problems (1.69; 95% CI, 1.18 to 2.42; P = 0.005) 12 months after admission.

## Discussion

Our studies provide a recent and unique portrait of selected health risk behaviors and mental health problems reported by detained boys. A key strength of the study lies in the fact that we were able to make direct comparisons with control groups. The control groups were not selected from a school–based population, but rather from a community–based population. Researchers in previous studies have encountered major problems when recruiting schools for health research, which can result in a poor response rate, various academic levels, and different geographical areas. This can introduce more biases and errors [16], [17]. Therefore, it is best to recruit control groups from a community–based population in the same region, which generally results in a higher response rate.

By contrasting the offenders with the community adolescents, the YRBS results show that young offenders are more likely to report risk behaviors, such as illegal drug use, sexual intercourse, suicide attempts, irregular diet, and physical fighting behaviors [18], and the logistic regression confirmed this result. These associations have all been well documented in Western countries and Asian countries [19], [20]. A survey of young offenders in the U.K. also showed that current drug use, sexual intercourse, physical fights, and suicidal attempts clustered in the groups [21]. This indicates that the risk behavior (e.g., drug use) probably contributes to related illegal behavior with a different magnitude.

Of note, although the proportion of tobacco use, alcohol use and high–risk cycling of young offenders ended to be higher than the control group, there was no significant difference in these characteristics between the two groups. The health risk behaviors in young offenders in this study were at levels similar to other studies on young offenders, but the levels in this study’s control group were higher than in control groups from American and European studies, which is similar to findings in other regions of China [19], [20], [22]. A large community–based study in China showed that 70.4% of male adolescents were lifetime alcohol users and 39.4% were current alcohol users. Tobacco use and high–risk cycling were also more common among senior adolescents (73.7% and 43.8%, respectively) [23]. Another study in China showed that the rate of alcohol and tobacco use had been increasing from 1998 to 2003, because drinking and smoking may be considered an important method of social communication. Cultural and socioeconomic differences may have contributed to the high prevalence rates in the control group relative to other countries [24].

We also found that a greater proportion of young offenders reported significantly higher scores for the 12 YSR outcomes than adolescents in the community sample at the first interview, which is similar to findings in other studies [25], [26]. In line with previous studies, the findings thus suggest that young offenders are at an increased risk of behavioral and mental health problems. The frequency of aggressive behavior, self-destructive/identity problems and externalizing problems were substantially closer to the controls 6 months after admission, which was also the case for delinquent behavior after 12 months. This reduction is probably due to the strict management and because many educational needs are being met [27]. All youths in detention centers must follow the rules and regulations to study, exercise, and work, which could have improved the above-mentioned mental health of these boys.

However, there were no obvious differences between the three interviews in the proportion of adolescents with social, thought, and attention problems or somatic complaints. However, the frequency of withdrawn, anxious or depressed and internalizing problems increased significantly 12 months later. Previous studies from various Western countries have revealed similar results. R Michael et al in Germany found the prevalence of attention-deficit hyperactivity disorder (ADHD) was significantly elevated in young adult prison inmates [28]. Another study in the U.K showed that the levels of depression and anxiety were high within 3 months of admission because no appropriate assessment or treatment had been offered [29]. This result provides evidence that detention centers may lead to or aggravate mental disorders because the adolescents are exposed to new traumas from the arrest and confinement. This conclusion must be drawn cautiously because larger samples would be needed to test how much rates of emotional problems are elevated.

Our results should be confirmed in other situations because there were some limitations in our research. First, only adolescents from Chongqing conducted the survey, and the sample may not represent all Chinese young offenders. Second, in collecting data, boys were asked to provide some retrospective information; hence, recall bias is unavoidable. Third, self report measures were used only and the absence of diagnostic categories was present. A final limitation of this study is a lack of information on detained adolescent females, and consequently, we do not know how gender affects these results [30], [31]. Thus, our results suggest that we need to pay closer attention to young offenders.

Our findings indicate that some risk behaviors probably contribute to related illegal behavior with a different magnitude, and moreover, that mental health need is not static but changes rapidly in different periods of detention. It seems that juvenile offenders in detention centers not only need primary care services but also need suitable psychological or psychiatric treatment for behavioral and emotional disorders. This might help policy–makers and researchers to incorporate prevention and treatment strategies to reduce risk behaviors of these adolescents and to prevent mental disorders.
